# Supplementary material for: Antifouling PVC Catheters by Gamma Radiation-Induced Zwitterionic Polymer Grafting
Source: Polymers (Basel). 2022 Mar 16;14(6):1185. doi: 10.3390/polym14061185 (PMC8950535; doi:10.3390/polym14061185)
Supplement: Supplementary file 1 [file polymers-14-01185-s001.zip › polymers-1624790-supplementary.pdf]

polymers

Supplementary material

Antifouling PVC Catheters by Gamma Radiation-Induced Zwitterionic Polymer Grafting

Lorena Duarte-Peña * and Emilio Bucio *

Departamento de Química de Radiaciones y Radioquímica, Instituto de Ciencias Nucleares, Universidad Nacional Autónoma de México, Circuito Exterior, Ciudad Universitaria, Ciudad de México 04510, Mexico
* Correspondence: lorena.duarte@correo.nucleares.unam.mx (L.D.-P.); ebucio@nucleares.unam.mx (E.B.)

1. BSA quantification: calibration curve

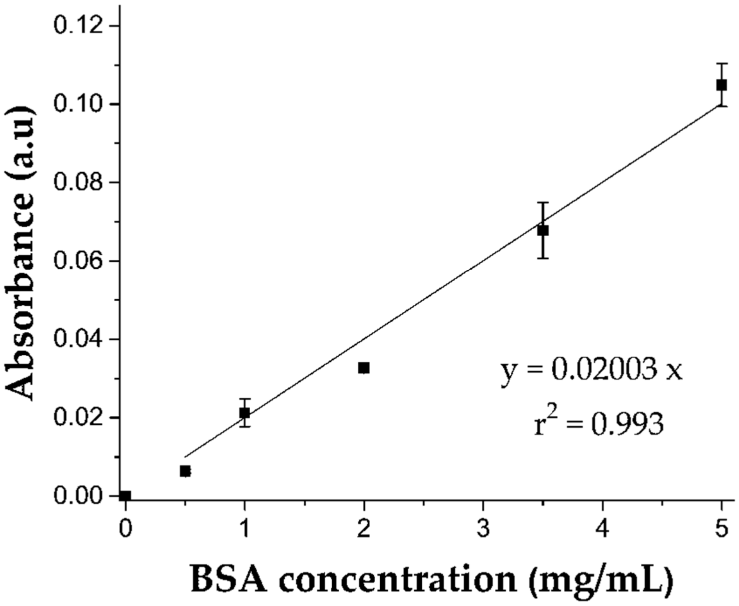

Figure S1. Calibration curve to BSA quantification (Abs 556 nm).

2. BSA quantification: working solution preparation

The working solution is a light green hue solution that consists of the 50 parts of solution A and 1 part of solution B.

Solution A: Dilute sodium bicarbonate (0.1 g), sodium carbonate dihydrate (2.0 g), sodium tartrate (dihydrate) (0.16 g), sodium hydroxide (0.4 g), sodium bicarbonate (0.95 g), in 100 mL of distilled water, adjusting the pH to 11.25 with NaOH.

Solution B: dilute copper sulfate (II) pentahydrate ($CuSO_4 \cdot 5H_2O$) to 4% (w/v) in distilled water.

3. Dose rate effect in the SBMA grafting: the t-significance contrast test

The correlation constant t between the two dose rates was calculated using the following equation:

$$t = \frac{(\bar{x}_1 - \bar{x}_2)}{s\sqrt{\frac{1}{n_1} + \frac{1}{n_2}}}$$

Where $\bar{x}_m$ is the average graft of each sample, n_m is the number of repetitions of each measurement and s is the pooled estimate of the standard deviation (σ), which is shown below:

$$s^2 = \frac{(n_1 - 1)\sigma_1^2 + (n_2 - 1)\sigma_2^2}{(n_1 + n_2 - 2)}$$

$(n_1 + n_2 - 2)$ indicate the freedom degrees of the comparison [1]. Table S1 shows the t-experimental value vs t critical value. t-experimental value is less than critical value, indicating that there is no significant difference among the values.

Table S1. t-significance contrast test.

Dose rate (kGy/h)	Freedom degrees	t-experimental value	t (P<0.05) critical value
5.4 vs. 13.0	4	1.20	2.78

Reference

1. Miller, J.N.; Miller, J.C. *Statistics and chemometrics for analytical chemistry*; Pearson/Prentice Hall, 2005; ISBN 9780131291928.
